# An Image-Free Opto-Mechanical System for Creating Virtual Environments and Imaging Neuronal Activity in Freely Moving *Caenorhabditis elegans*

**DOI:** 10.1371/journal.pone.0024666

**Published:** 2011-09-28

**Authors:** Serge Faumont, Gary Rondeau, Tod R. Thiele, Kristy J. Lawton, Kathryn E. McCormick, Matthew Sottile, Oliver Griesbeck, Ellie S. Heckscher, William M. Roberts, Chris Q. Doe, Shawn R. Lockery

**Affiliations:** 1 Institute of Neuroscience, University of Oregon, Eugene, Oregon, United States of America; 2 Applied Scientific Instrumentation, Eugene, Oregon, United States of America; 3 University of California San Francisco, San Francisco, California, United States of America; 4 Department of Neurobiology and Behavior, Cornell University, Ithaca, New York, United States of America; 5 Galois Inc., Portland, Oregon, United States of America; 6 Max-Planck-Institute of Neurobiology, Martinsried, Germany; 7 Howard Hughes Medical Institute, Institute of Neuroscience, Institute of Molecular Biology, University of Oregon, Eugene, Oregon, United States of America; Harvard University, United States of America

## Abstract

Non-invasive recording in untethered animals is arguably the ultimate step in the analysis of neuronal function, but such recordings remain elusive. To address this problem, we devised a system that tracks neuron-sized fluorescent targets in real time. The system can be used to create virtual environments by optogenetic activation of sensory neurons, or to image activity in identified neurons at high magnification. By recording activity in neurons of freely moving *C. elegans*, we tested the long-standing hypothesis that forward and reverse locomotion are generated by distinct neuronal circuits. Surprisingly, we found motor neurons that are active during both types of locomotion, suggesting a new model of locomotion control in *C. elegans*. These results emphasize the importance of recording neuronal activity in freely moving animals and significantly expand the potential of imaging techniques by providing a mean to stabilize fluorescent targets.

## Introduction

The ability to record the activity of particular neurons non-invasively in untethered, freely moving animals would greatly accelerate studies of the neuronal basis of behavior, but such recordings remain generally elusive. Head-mounted telemetry systems have made single unit recordings possible in untethered mammals and birds [1], but this approach is invasive and the identities of recorded neurons are often uncertain. Truly non-invasive recordings have been made in freely moving zebrafish larvae by means of an activity-dependent bioluminescent probe that is transgenically expressed in target neurons and monitored by a wide-field photodetector [2]. However, this approach reports the summed activity of all neurons expressing the probe, making it impossible to assign signals to individual neurons. Thus, current technology has allowed only limited progress toward recording the activity of identified neurons in freely moving animals.

To record from identified neurons in untethered animals it is usually necessary to genetically target an optical probe to known subsets of neurons. Such recordings have been achieved from groups of neurons, or spatially isolated single neurons, in freely moving *Caenorhabditis elegans*. This was done by periodically recentering the target in the field of view either manually [3] or by means of image processing software that controlled a motorized stage [4], an approach developed more than a decade ago for behavioral tracking experiments [5]. A fundamental limitation of this method is that under conditions of normal locomotion, the target neuron moves large distances during the time taken to process the neuronal image and recenter the stage. As a result, the neuron escapes the field of view unless a wide-field microscope objective is used. However, the magnification of such objectives (≤20×) is insufficient to resolve the somata of individual *C. elegans* neurons unless they happen to be far apart. Thus, the image processing approach is not a general method for recording from neurons of interest in freely moving *C. elegans* and other model organisms.

To develop a general method for recording from identified neurons in freely moving *C. elegans* and other model organisms, we devised an image-free, opto-mechanical system that recenters fluorescent targets moving at speeds of up to 500 µm/s. Importantly, the new system is compatible with high performance microscope objectives (63×–100×, 1.3–1.4 N.A.), making it possible to resolve densely packed neurons in freely moving animals for the first time. The system is based on a high-speed feedback loop between the location of the target in the field of view and compensating movements of the microscope stage, reducing the latency of recentering movements by a factor of up to 40 relative to previous systems [4]. The recentering system can be used in two modes. In stimulation mode, the instantaneous position of the target, measured in stage coordinates, is used to optogenetically activate identified sensory neurons to induce fictive perceptions. In recording mode, the system is used to keep the target neuron in the field of view of a 63× microscope objective.

Using the new system, we tested the long-standing hypothesis that forward and reverse locomotion are generated by distinct neuronal circuits in *C. elegans* [6]. These circuits are comprised of separate populations of motor neurons specifically required for forward and reverse locomotion, together with their anatomically associated pre-motor interneurons. Surprisingly, in each circuit we found neurons that are active during both forward and reverse locomotion. This finding suggests that the control of locomotion in *C. elegans* may be less modular than previously thought and emphasizes the importance of making simultaneous recordings of neuronal activity and behavior in freely moving animals when attempting to deduce the function of neural circuits.

## Results

### Image-free, opto-mechanical recentering of rapidly moving fluorescent targets

Genetically encoded probes of neuronal activity such as cameleon [7], TN-XL [8], TN-XXL [9], and GCaMP [10] have numerous advantages for recording non-invasively from neurons in widely studied model organisms such as *C. elegans* and *Drosophila* larvae. However, resolving the small, densely packed neurons of these organisms requires the use of high performance microscope objectives (63×–100×, 1.3–1.4 N.A.). Unfortunately, the field of view of such objectives (≤150 µm×≤200 µm) is small relative to the speed of these organisms, as worms and fly larvae travel at speeds up to 500 µm/s and 1000 µm/s, respectively [5], [11] (see also below). When standard optics for ratiometric imaging are used to track objects moving at such speeds, a centered neuron can escape the field of view in less than 150 ms, an interval too brief for meaningful data to be acquired.

The obvious solution to the problem of target escape is to automatically recenter the object. The conventional way of doing this is to capture a frame and use digital image processing to locate the object, compute its eccentricity, and command a motorized microscope stage to make a compensating movement [4]. This approach, however, currently has significant limitations when applied to neuronal targets. In its current instantiation, it operates at rate of 6.7 frames/s which leaves large gaps in the data. It also results in substantial blurring because the speed of the organism is high relative to the size of the neurons and the exposure time required to image them (25–50 ms). For example, when traveling at 500 µm/s, the 2 µm soma of a *C. elegans* neuron moves more than 6–13 cell diameters while the camera is acquiring a frame. Blurring of this order is a serious problem because it distributes the target neuron's photons over an area many times larger than the neuron itself, thereby depressing the signal-to-noise ratio at each pixel in the neuron's path by a factor approximately equal to the number of cell diameters traveled during a frame.

**Figure 1:**
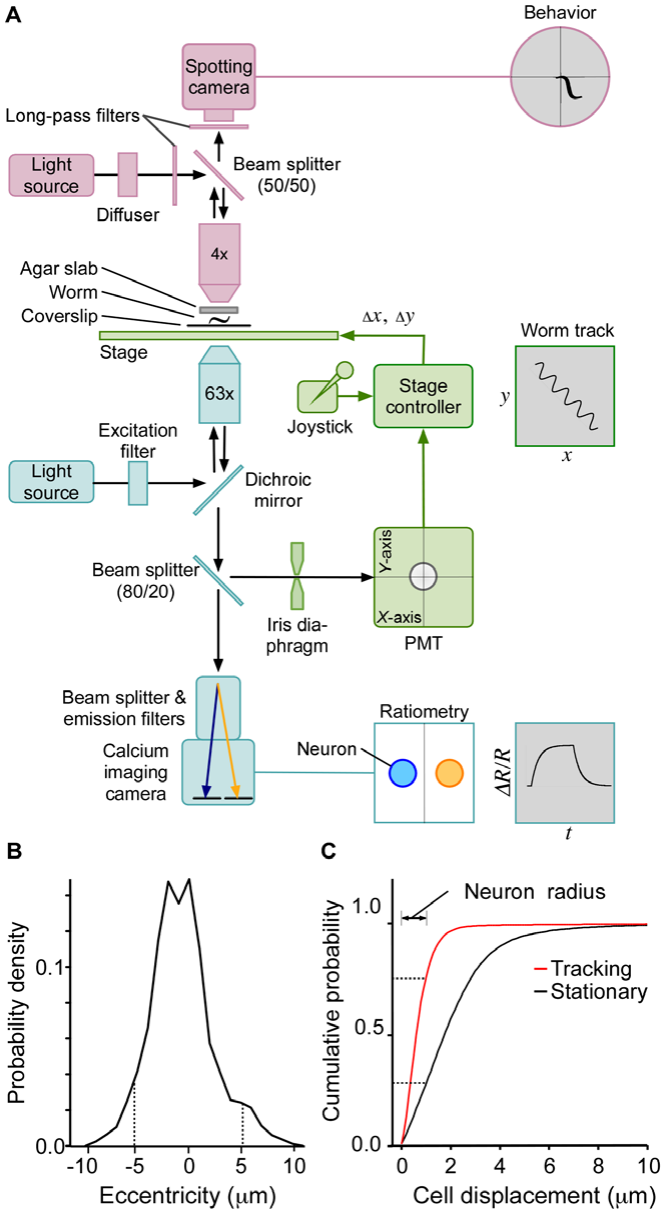
Tracking system. **A**. Schematic diagram of the recording system. Three subsystems are indicated: Ratiometric calcium imaging (blue), behavior imaging (red), and image recentering (green). Gray objects show the output of each subsystem. **B**. Performance of the image recentering subsystem, as illustrated by the probability density of radial eccentricities for a single neuron in a freely moving animal. Dashed lines show the region containing 95% of the data. **C**. Performance of the image recentering subsystem, as illustrated by the cumulative probability of cell displacement between contiguous frames in tracking mode (green line) and for a stationary stage (black line). Data for a stationary stage were predicted based on the animal's speed at the time the frame was taken. Dotted lines indicate the cumulative probabilities for a displacement of one neuron radius in the tracking and stationary modes. Measurements were obtained from the same data set as in **B**.

To address these limitations, we developed an image-free, opto-mechanical system in which a dedicated circuitry continuously recenters the fluorescent target while neuronal activity and behavior are being recorded (Fig. 1, S2; see Methods). This was accomplished by diverting 20% of the light from a 63× objective to a four-quadrant photomultiplier tube (PMT) by means of a beam splitter or, in some experiments, a dichroic mirror. The four analog intensity signals from the PMT were fed into a motorized-stage controller with circuitry dedicated to computing the degree of skew in the distribution of light falling on the PMT. Skew values continuously regulated the speed of servo motors in a motorized stage via a gain factor that could be adjusted by the user according to the speed of the target.

The recentering system was integrated with an inverted compound microscope. This instrument was fitted with standard optics for FRET-based, dual-wavelength ratiometric calcium imaging (Fig. 1a), and relative somatic calcium concentration served as a proxy for neuronal activity. Whole-animal behavior was recorded in infrared light by means of a wide-field, low-power objective (4×) attached to a spotting camera mounted above the stage. The fully integrated system yielded three synchronized data streams: the intensity ratio of the two wavelengths of the calcium probe (20–40 frames/s), a video of whole-animal behavior (22 frames/s), and the *x-y* position of the target (20–50 samples/s) from which the animal's velocity and spatial trajectory can be determined in real time.

Good measures of a tracking performance are latency from signal capture to stage movement, the precision of recentering movements, and maximum target speed. For systems based on image processing, minimum latency with commercially available components is approximately 80 ms (50 ms exposure time, 15 ms read time, and 15 ms for image processing and data transmission to the motion controller). By contrast, direct measurements showed that the latency of our system is approximately 2 ms (Fig. S1), representing a 40-fold improvement over the expected latency in optimal image-based approaches. To measure recentering precision, we expressed cameleon (YC3.6) under the control of the *cex-1* promoter, which uniquely labels the head interneuron RIM. We then measured the standard deviation of eccentricity values obtained on a frame-by-frame basis when RIM was tracked in moving animals (top speed 188±15 µm/s, 12 animals, Fig. 1b). Precision, defined as the width of the region of the eccentricity distribution that contained 95% of its values, was ±5.2 µm (Fig. 1b). In tests of maximum target speed, we found the system to be capable of following larval nematodes crawling in a fluid-filled matrix [12] at speeds of up to 500 µm/s (Movie S1), a value 3 times higher than the average speed of a worm on an agarose surface [5]. We also found the system to be capable of following *Drosophila* larvae moving at similar speeds, indicating that it is applicable to other model organisms (Movie S2). Taken together, the performance tests showed that our system yields a general method for tracking rapidly moving targets viewed with high magnification microscope objectives.

The system's short response latency, together with its continuous regulation of stage speed, suggested that blurring should be significantly reduced relative to the image processing approach. We assessed blurring by measuring the frame-to-frame displacement of the tracked neuron in the field of view of the calcium imaging camera (RIM, Fig. 1c). Using a displacement of one cell radius (1 µm) as a cutoff to distinguish between blurred and unblurred images, we found that 76% of displacement values were below the cutoff. Using the same set of frames, we computed the theoretical frame-to-frame displacement that would have occurred if the stage were stationary during image acquisition, as it would be in a recentering system based on image processing. To compute this displacement, we simply multiplied the frame duration by the velocity of the animal. We found that now only 29% of displacements were below the cutoff. This analysis indicates that blurring was substantially reduced by the recentering system.

### Osmotic avoidance responses in virtual environments

**Figure 2:**
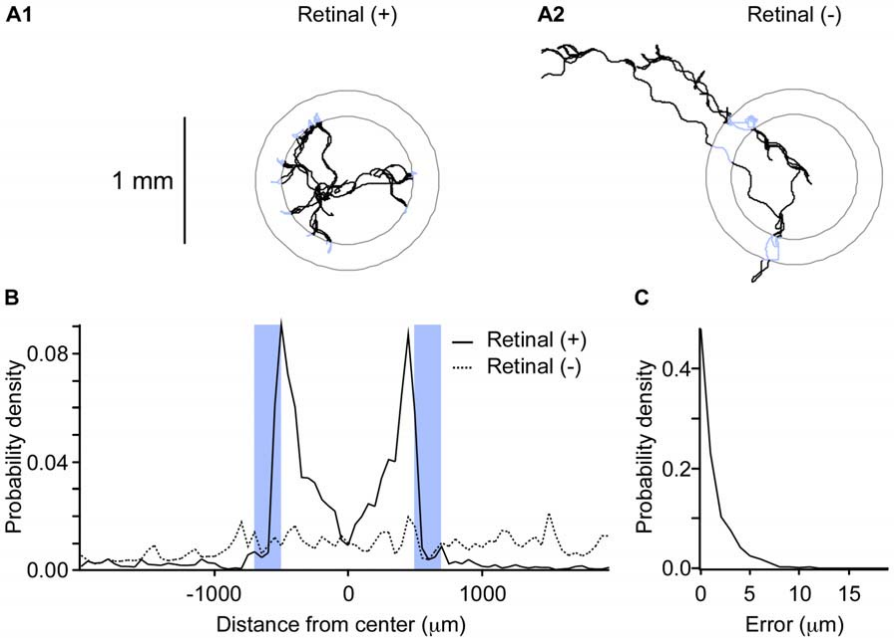
Osmotic avoidance responses in a virtual environment. Channelrhodopsin-2 was specifically expressed in the osmo-sensitive neuron class ASH. Animals were tracked via a small cluster of neurons expressing a red fluorescent protein located in the head. ASH was photoactivated whenever the animal's head entered an annular region with outside and inside diameters of 1.4 and 1.0 mm, respectively. **A**. Representative tracks of animals raised in presence (**A1**) or absence (**A2**) of the co-factor retinal; blue segments indicate photoactivation. **B**. Probability density of the distance from the center of the annulus for 25 retinal (+) and retinal (−) animals. The blue bars indicate the photoactivation zone. **C**. Probability density of spatial error, defined as the distance between the tracking target and the nearest annulus border at the onset of photoactivation.

The level of spatial precision found in performance tests, coupled with the high sampling rate of *x-y* position, suggested that it should be possible to create high resolution virtual environments using the new system. To demonstrate this mode of use, we used channelrhodopsin (ChR2) [13] activation of sensory neurons to simulate a classical behavioral screen [14] in which worms are placed in the center of an annulus of high osmolarity fluid, from which they normally recoil (Fig. 2). ChR2 was expressed specifically in the osmo-sensitive neuron class ASH, which has been shown to mediate avoidance of life-threatening regions of high osmolarity [15], [16]. The recentering system was programmed to stimulate ASH by turning on a blue light to activate ChR2 whenever the worm's head entered an annulus-shaped region of space. As expected, worms fed retinal, the essential co-factor for ChR2, avoided the annulus as though it were a region of high osmolarity, remaining trapped in the center (Fig. 2A1, B), whereas unfed control worms did not (Fig. 2 A2, B). In addition, we found that the light was triggered with a spatial accuracy on the order of microns (Fig. 2C). This experiment provides proof-of-concept for creating high resolution virtual environments for exploring the neuronal control of behavior in freely crawling organisms.

### Dedicated circuits for locomotion control in *C. elegans*

**Figure 3:**
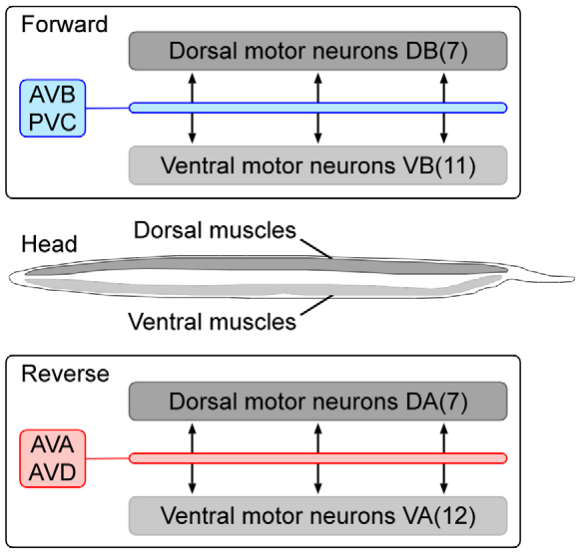
The dedicated circuit model of locomotion control in *C. elegans*. The forward circuit is composed of the command neuron classes AVB and PVC together with dorsal (D) and ventral (V) classes of B-type motor neurons. The reverse circuit is composed of the command neuron classes AVA and AVD together with dorsal and ventral classes of A-type motor neurons. Each class of command neuron consists of a left-right pair of monopolar neurons whose process runs the length of the animal, forming presynaptic connections en passant with the motor neurons. The number of motor neurons in each class is shown in parentheses. Motor neurons are distributed evenly along the length of the animal.

Non-invasive recordings from neurons in moving animals are particularly well suited to studies of locomotion. On an agar surface, nematodes crawl on their sides, generating thrust by means of sinuous dorsal-ventral undulations that travel head-to-tail during forward locomotion and tail-to-head during reverse locomotion. The periodic contractions of the body wall muscles that underlie these movements are thought to be produced by membrane potential oscillations in two main types of excitatory motor neurons distributed along the length of the body (Fig. 3): the dorsal and ventral “A type” motor neurons (DA, VA), and the dorsal and ventral “B type” motor neurons (DB, VB). Anatomical reconstructions show that A and B type motor neurons are innervated predominantly by distinct classes of pre-motor interneurons, referred to in *C. elegans* as “command neurons” (Fig. 3).

In what we shall refer to as the “dedicated circuit model” of locomotion control in *C. elegans*, there are separate, functionally distinct circuits for forward and reverse locomotion [6], [17], [18], [19]. In support of the model, ablation of B-type motor neurons specifically impairs forward locomotion, whereas ablation of the command neurons that innervate them (AVB, PVC) specifically renders forward locomotion uncoordinated; thus, these three neuron types are hypothesized to comprise the forward circuit. Conversely, ablation of A-type motor neurons specifically eliminates reverse locomotion, whereas ablation of the command neurons that innervate them (AVA, AVD) specifically renders reverse locomotion uncoordinated; thus, these three neuron types are hypothesized to comprise the reverse circuit [6]. Forward and reverse undulations are presumed to be generated by antiphasic oscillations in the dorsal and ventral motor neurons of each circuit, but how these oscillations are generated is unclear.

Neurophysiological evidence for the dedicated circuit model is limited. There are no recordings of locomotory command neurons or motor neurons in freely moving worms, with the exception of one study that reported increased activity during reverse locomotion in a cluster of interneurons that contains the reverse command neuron AVA [4]. Calcium imaging experiments in semi-restrained animals are only partially consistent with the dedicated circuit model: AVA and the A-type motor neurons are activated during reverse locomotion, whereas the B-type motor neurons are activated during forward locomotion [20], [21]. Surprisingly, however, motor neuron activity in semi-restrained animals does not oscillate, which raises the question of whether these results can be generalized to freely moving animals. To expand and clarify the evidence for the dedicated circuit model, we used our recentering system to image calcium transients in command neurons and motor neurons during locomotion in freely crawling animals.

### Simultaneous recordings of command neuron activity and behavior support the dedicated circuit model

#### AVA

**Figure 4:**
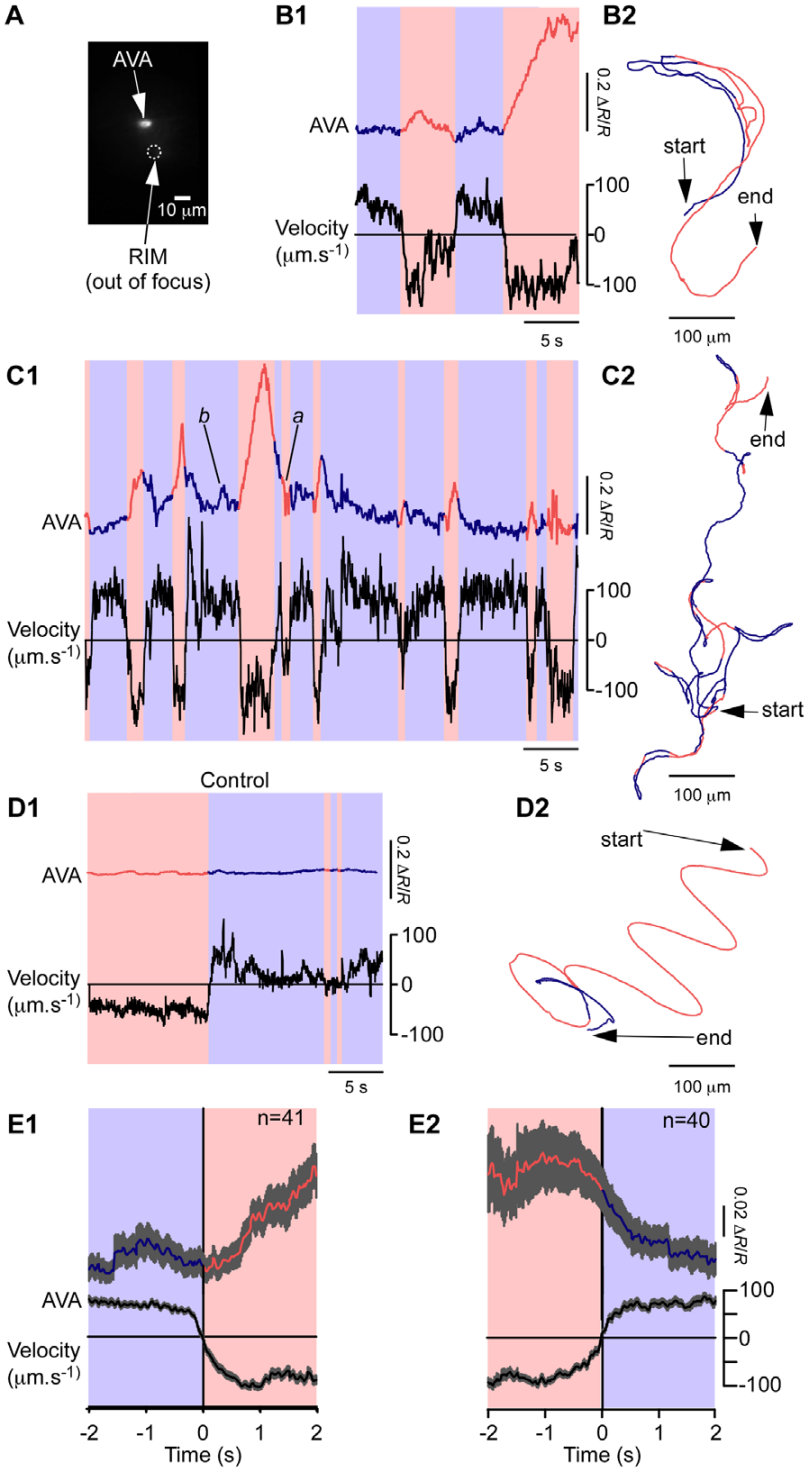
Simultaneous recordings of the activity of the command neuron AVA and the locomotory behavior of freely moving animals. **A**. Fluorescence image from the calcium imaging camera showing the position of AVA; the position of the interneuron RIM, which lies in a different focal plane, is indicated by the circle. **B1**, **C1**. Representative time courses of AVA emission ratio (upper trace) and velocity (lower trace) in two different animals. The colors of the emission ratio trace and background indicate the direction of locomotion (blue, forward; red, reverse). **B2**, **C2**. The *x*-*y* trajectory of the neurons recorded in **B1** and **C1**; colors as above. **D1**, **D2**.Control for movement artifacts in AVA neurons expressing a calcium insensitive fluorescent protein (GFP). Emission ratio, velocity, and *x*-*y* trajectory are plotted as in **B** and **C**. **E**. Ensemble averages of emission ratio and velocity during changes in the direction of locomotion (**E1**, forward to reverse; **E2** reverse to forward). Grey shading represents ±SEM.

The dedicated circuit model predicts that reverse command neurons such as AVA neurons should be active during reverse locomotion and inactive during forward locomotion, but this prediction has yet to be tested in freely moving worms at high magnification. To record from AVA, we expressed the ratiometric calcium sensor TN-XL [8] under the control of the *nmr-1* promoter, the same promoter used in the earlier studies [4]. This promoter is expressed in six bilaterally symmetric neuron classes (one left and right neuron per class), three of which occur in a cluster containing the command neurons AVA and AVE, and the interneuron RIM. Despite the clustering of *nmr-1* positive neurons, the system's high performance objective allowed us to identify the AVA cell body simply by the stereotypical position of its soma and axon, as other *nmr-1* positive neurons were either spatially resolvable or in a different focal plane (Fig. 4a, Movie S3). The emission ratio in AVA increased and remained above baseline during reverse movement and decreased, or remained low, during forward movement (Fig. 4b1, c1). As in previous recordings [4], [21], AVA calcium concentration did not appear to oscillate during bouts of either forward or reverse locomotion that were long enough to support at least one complete undulation cycle (Fig. 4b2, c2). The absence of such oscillations implies that AVA neurons are not part of the presumptive pattern generator for locomotion in *C. elegans*. As a control for movement artifacts, we recorded from a strain in which GFP was expressed in the same set of neurons (Fig. 4d1), exploiting the calcium insensitivity of GFP and the breadth of its emission spectrum, which allows for pseudo cyan-yellow ratiometry. AVA ratio traces in control animals were flat (44 reversals in 9 animals), indicating that movement artifacts did not contribute significantly to recorded calcium transients. Taken together, these results indicate that our system is capable of imaging single neurons in freely crawling worms.

We quantitatively assessed the degree of correspondence between AVA calcium transients and individual bouts of reversal behavior. The great majority of reversals (36/40) coincided with a readily detectable calcium transient. In the few instances in which a reversal occurred in the absence of a calcium transient, the reversal was brief, lasting less than 2 s (e.g., Fig. 4c1, event *a*). Conversely, the majority of apparent calcium transients (40/45) coincided with a reversal bout. In the few instances in which a calcium transient occurred in the absence of a reversal, the transient was small, with Δ*R*/*R*<0.1 (e.g., Fig. 4c1, event *b*). Such exceptions may reflect signal-to-noise limitations inherent in the use of calcium probes in freely moving nematodes. Thus, the correspondence between ratio changes and changes in the direction of locomotion is robust. In a separate analysis, we compared the time course of ratio changes with the time course of locomotion velocity by computing ensemble averages of ratio and velocity traces (Fig. 4e). Traces were aligned to the time of the zero crossing of the velocity axis either during forward-to-reverse transitions (F–R; Fig. 4e1) or during reverse-to-forward transitions (R–F; Fig. 4e2). This analysis showed that the average ratio increased in association with F-R transitions, and decreased in association with R-F transitions. Based on the correspondence between calcium transients and reversal events, together with the observed associations between ratio changes and changes in the direction of locomotion, we conclude that the pattern of activity in AVA is consistent with the dedicated circuit model.

We noted, however, that the time course of average ratio changes in command neurons did not precisely mirror the time course of average velocity. For example, in the case of both F-R and R-F transitions, the ratio continued to rise or fall, respectively, even after velocity had reached its new steady state level, indicating that changes in ratio were somewhat slower than changes in velocity. A simple explanation for the discrepancy is that somatic calcium concentration may fluctuate more slowly than the electrophysiological events at the synaptic sites where motor neuron activity is regulated, which in command neurons are hundreds of microns distant from the soma; this discrepancy might be exacerbated by the slow response typical of genetically encoded calcium probes [22].

#### AVB

**Figure 5:**
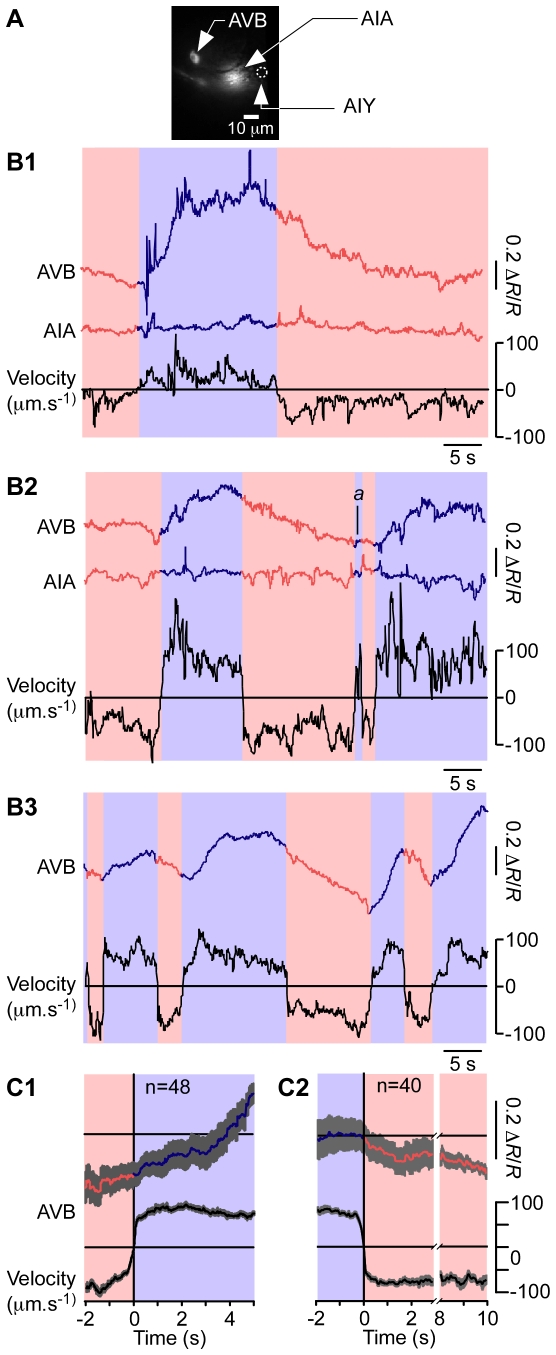
Simultaneous recordings of the activity of the command neuron AVB, the interneuron AIA, and the locomotory behavior of freely moving animals. **A**. Fluorescence image from the calcium imaging camera showing the positions of the two neurons. **B1–B3**. Representative time courses of AVB (**B1–3**) and AIA (**B1–2**) emission ratios (upper traces) and velocity (lower trace) in three different animals. The colors of the emission ratio trace and background indicate the direction of locomotion (blue, forward; red, reverse). The AIA recordings serve as internal controls for movement artifacts. **C1**, **C2**. Ensemble averages of AVB emission ratio and velocity during changes in the direction of locomotion (**C1**, reverse to forward; **C2** forward to reverse). Note that the time axis in **C2** was modified to reflect the slow time course of the emission ratio. Grey shading represents ±SEM.

The dedicated circuit model predicts that forward command neurons such as AVB neurons should be active during forward locomotion and inactive during reverse locomotion. To record from AVB we expressed the ratiometric calcium sensor cameleon (YC3.60) under the *sra-11* promoter, which is expressed in the interneuron classes AVB, AIA, and AIY (Fig. 5a, Movie S4). Despite the close proximity of these neurons, we were again able to resolve and identify all three neurons. The AVB emission ratio increased and remained above baseline during forward movement and decreased or remained low during reverse movement (Fig. 5b; *n* = 48 forward bouts in 13 animals). AVB calcium concentration did not appear to oscillate suggesting that, like AVA, this command neuron class is not directly involved in pattern generation.

The fact that we were able to resolve densely packed neurons in freely moving animals afforded an internal control for possible movement artifacts in the AVB recordings. In cases in which the two cell bodies were in the same focal plane (5 out of 13 animals), we were able record from AVB and AIA neurons simultaneously. Although AIA is not considered a locomotory command neuron, ablating it increases the frequency of reversal bouts under certain sensory conditions [23]. The AIA traces were essentially flat (Fig. 5 b1–2), suggesting that AIA was neither active and nor subject to movement artifacts. Because the somata of AIA and AVB are separated by only 30 µm, their motions are tightly coupled. Thus, the absence of signals in AIA indicates that signals seen in AVB neurons reflect changes in calcium concentration rather than movement of the neuron. This experiment demonstrates the ability of our system to record from multiple neurons simultaneously.

We next assessed the degree of correspondence between AVB calcium transients and individual bouts of forward locomotion. Most forward bouts (43/47) coincided with a readily detectable calcium transient. In the few instances in which an episode of forward locomotion occurred in the absence of a calcium transient, the episode was brief, lasting less than 2 s (e.g., Fig. 5b2, event *a*). AVB calcium transients in the absence of forward locomotion were not observed. As in the case of AVA, we also compared the time course of ratio changes in AVB with the time course of locomotion velocity by computing ensemble averages of ratio and velocity traces (Fig. 5c). This analysis showed that the average ratio increased in association with R–F transitions, and slowly decreased in association with F–R transitions, indicating that the relationship between calcium transients and direction of locomotion is robust. Based on the correspondence between calcium transients and reversal events, together with the observed associations between ratio changes and changes in the direction of locomotion, we conclude that the pattern of activity in AVB is consistent with the dedicated circuit model.

### Simultaneous recordings of motor neuron activity and behavior fail to support the dedicated circuit model

**Figure 6:**
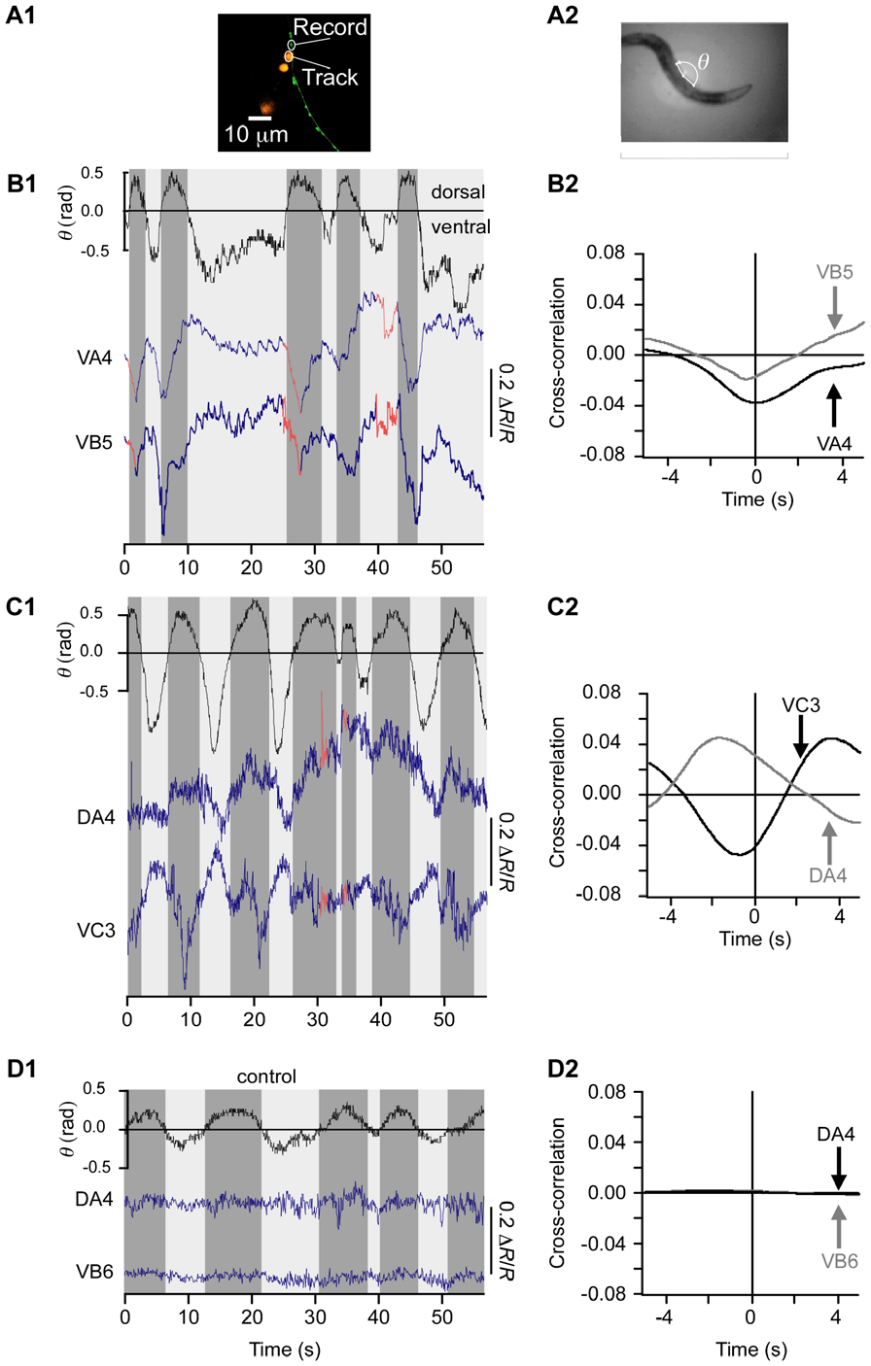
Simultaneous recordings of the activity of the motor neurons and the locomotory behavior of freely moving animals. **A1**. Fluorescence image showing the relative positions of neurons (green) and coelomocytes (red) used as proxy tracking targets. The latter were labeled with the fluorescent protein dsRed. **A2**. An image from the spotting camera illustrating the analysis of dorsoventral undulations. Undulations were quantified by the angle *θ* formed by the line segments between three points located on the outline of the animal, as shown. Positive and negative angles correspond to dorsal and ventral bends, respectively. **B1**, **C1**. Representative time courses of bending angle (upper trace) and emission ratio in the indicated motor neurons (lower traces) in two different animals. The colors of the emission ratio trace indicate the direction of locomotion (blue, forward; red, reverse); the color of the background indicates the direction of the body bend (dark gray, dorsal; light gray, ventral). **B2**, **C2**. Cross-correlations between bending angle and emission ratio in the recordings shown in **B1** and **c1**. Trace color corresponds to motor neuron identity as indicated. **D**. Control for movement artifacts in motor neurons expressing a calcium insensitive fluorescent protein (GFP). Bending angle, emission ratio, and cross-correlations plotted as above.

The motor neurons of the body wall muscles in *C. elegans* are particularly challenging to record from because their somata are smaller than those of most other *C. elegans* neurons. Additionally, when fluorescently labeled, their overlapping processes form an elongated target along which a tracking system tends to drift. To address this problem, we genetically introduced isolated punctate proxy targets that emitted at longer wavelengths than the ratiometric calcium probes. This was done by expressing the fluorescent protein dsRed under the control of the *unc-122* promotor, which is specifically expressed in a non-neuronal cell type that is sparsely distributed throughout the animal, often occurring in close proximity to the target motor neurons (Fig. 6a1). Exploiting the fact that dsRed is marginally excited by the blue light used to excite the calcium probes, we replaced the 80/20 beam splitter in the recentering system (Fig. 1a) with a short pass dichroic mirror thereby specifically directing dsRed emission to the PMT. This enabled the system to record motor neurons by recentering the nearest proxy target.

To demonstrate the viability and utility of the proxy target method, we recorded from A-type and B-type motor neurons. This was done by expressing the ratiometric calcium sensor TN-XXL under the control of the *unc-17* promotor, which is expressed in all A and B type motor neurons. The *unc-17* promoter is also expressed in the AS and VC motor neurons, which innervate dorsal and ventral muscles, respectively. Ratio traces in motor neurons exhibited robust oscillations (Fig. 6b–c; Table S1, Movie S5). To ascertain whether these oscillations were correlated with undulations, we measured the bending angle *θ* in the vicinity of the recording site as shown in Fig. 6a2 (see Methods). We then computed cross-correlations between *θ*(*t*) and Δ*R/R* (Fig. 6b2–c2). We found that motor neurons innervating ventral muscle (VA4, VB5, and VC3) oscillated in phase with ventral bends, whereas motor neurons innervating dorsal muscle (DA4) oscillated in phase with dorsal bends. As a control for movement artifacts, we recorded from a strain in which GFP was expressed in the same set of motor neurons (Fig. 6d1). In 33 cells in 19 animals, ratio traces were not correlated with bending angle (Fig. 6d2) arguing against movement artifacts. Additional evidence against movement artifacts is provided by the fact that we regularly observed out-of-phase motor neuron activity in nearby motor neurons recorded simultaneously (Fig. 6c1–2). Overall, oscillations in each type of motor neuron were consistent with a role in producing the contractions observed on the side of the worm innervated by that motor neuron. As noted above, oscillations are absent when motor neurons are recorded in semi-restrained animals [20]. A possible explanation for this difference is that the body bends in the presence of mechanical constraints are not representative of undulations in freely moving animals because of the disruption of normal proprioceptive inputs to the motor neurons.

The dedicated circuit model predicts that A-type and B-type motor neurons should be active only during reverse and forward locomotion, respectively. We therefore expected the ratio traces in motor neurons for movement in a given direction to be flat when the animal was moving in the direction normally associated with the other type of motor neuron. Surprisingly, however, traces from A-type motor neurons were not flat but instead clearly oscillated during forward locomotion (Fig. 6, b1 and c1). Similar A-type motor neuron activity was observed in all recordings that contained transitions from reverse to forward locomotion (9 neurons in 9 animals). Analogously, traces from B-type motor neurons during reverse locomotion were not flat during reverse locomotion (Fig. 6b1, t = 0 s, t = 26 s, and t = 40 s). Similar B-type motor neuron activity was observed in all recordings that contained transitions from forward to reverse locomotion (8 neurons in 8 animals). We conclude that motor neuron recordings in freely moving animals contradict the dedicated circuit model.

## Discussion

To address current limitations in non-invasive recordings from identified neurons in moving animals, we devised an image-free, opto-mechanical system that recenters rapidly moving fluorescent targets. The new system represents a significant advance over previous approaches in four key respects. First, it is compatible with the high performance microscope objectives (63×, 1.4 NA) required to resolve minute, densely packed neurons. Second, the target is recentered via continuous, short latency stage movements. This feature enhances signal to noise ratio by minimizing blur. Third, the recentering mechanism tolerates relatively large changes in target position along the *z*-axis. This feature arises because the light falling on the PMT is not formed into an image, which could go out of focus and become lost. Fourth, the recentering mechanism does not utilize the images obtained for recording neuronal activity. This feature allows the frame rate and exposure time for calcium imaging to be optimized without adverse effects on the latency and precision of recentering. This feature also makes it possible to track proxy targets that emit at wavelengths that differ from those of the optogenetic calcium probe, thereby minimizing interference between tracking and recording targets. The use of proxy targets is a general method that could also be used for recentering any target that is undersized, dim, elongated, or otherwise unsuitable for recentering directly.

We have demonstrated that the recentering system can be used to create high resolution virtual environments by linking optogenetic photostimulation of sensory neurons to the *x-y* position of the tracking target with great precision. We used this mode to replicate a classical behavioral screen based on withdrawal responses from an annulus of high osmolarity fluid. By photostimulating neurons of other sensory modalities, this approach could be used to simulate other types of virtual environments: chemical or thermal gradients could be simulated by photoactivating chemosensory neurons or thermosensory neurons at intensities that depend on the worm's position and velocity; food patches could be presented by photoactivating aminergic neurons involved in the detection of food [24] and virtually textured environments could be created by photostimulating mechanosensory neurons in microscopic spatial patterns [25]. Importantly, because these environments are virtual, their shape and temporal dynamics could be experimentally manipulated in ways that transcend our limited ability to manipulate physical properties of the environment on the scale of microns and milliseconds.

When operated in recording mode, the recentering system provided new insights into the relationship between command neuron activity and locomotion in *C. elegans*. Analysis of undulatory locomotion in other organisms reveals three functional classes of interneurons [26], [27]: trigger neurons which are required only to initiate locomotion, gating neurons which are required to initiate and maintain locomotion, and oscillator neurons which are responsible for generating the rhythmic motor pattern. AVB and AVA neurons clearly function as gating neurons in that their activity is coextensive with locomotion in the forward and reverse direction, respectively, and neither type of neuron exhibits oscillations. Additionally, with allowances for the temporal delays inherent in calcium imaging, the activity of the command neurons appears consistent with the dedicated circuit model. However, because emission ratio is at best a relative measure of neuronal activity, we do not exclude the possibility that basal synaptic release from command neurons in their deactivated state contributes to locomotion.

Our recordings from motor neurons in freely moving animals provide new insights into the relationship between motor neuron activity and locomotion *C. elegans*. In particular, they suggest that the dedicated circuit model may be an oversimplification in. Such a model predicts that the A-type motor neurons of the reverse module should be inactive during forward locomotion whereas the B-type motor neurons of the forward module should be inactive during reverse locomotion. This appears not to be the case, however, as both types of motor neurons undergo strong modulations in activity during dorsoventral undulations associated with locomotion in the complementary direction. The proximal cause of motor neuron activity during the complementary behavior remains to be elucidated. However, both types of motor neurons are postsynaptic to the stretch receptor neuron DVA which extends along the length of the animal [28]. Additionally, body wall motor neurons in *C. elegans* may have intrinsic stretch receptor functionality, as has been hypothesized based on anatomical specializations of their distal neurites [29]. We therefore propose a model in which motor neurons are entrained by proprioceptive inputs during movement in the complementary direction. The failure to observe oscillations in motor neurons in semi-restrained animals might be explained by abnormalities in proprioception caused by restraint [20]. The new model appears to be at odds with the ablations showing that A-type and B-type motor neurons are not required for locomotion in the complementary direction [6]. However, those ablations were performed in larval animals whose motor circuitry differs from its adult form. Moreover, ablation effects were assessed without the benefit of quantitative analysis of changes in waveform. The proposed model could be tested by quantitative assessment of ablations or optogenetic manipulation of motor neurons in adult animals [30], [31].

The new system, when coupled with previous techniques [30], [31], sets the stage for a comprehensive analysis of the neuronal correlates of behavior in freely moving *C. elegans*. Although in this paper we focused on command neurons and motor neurons of the locomotion circuitry, it is possible in principle to record from any *C. elegans* neuron. Promoters have already been identified for key neurons in almost all *C. elegans* behaviors including most spatial orientation behaviors (chemotaxis, thermotaxis, aerotaxis), several forms of escape, foraging, feeding, egg laying, and mating. Although much can be learned by recording from one or two neurons at a time, to rigorously investigate the function of neural circuits it will be essential to record simultaneously from many neurons. At present, multineuron recordings in *C. elegans* are limited to neurons that lie in the same focal plane. This limitation could be addressed by combining our recentering system with *z*-axis scanning. Recording simultaneously from neurons in widely separated parts of the body will be more difficult, but fortunately more than 70% of all *C. elegans* neuron types are located in the head ganglia, which can be imaged in a single field of view.

The recentering methodology can be adapted to investigations of other genetically tractable, transparent animals such as the larvae of the fruit fly (*Drosophila melanogaster*) and zebrafish (*Danio rerio*). Indeed, we have shown that *Drosophila* larvae can be tracked and, using a motorized stage with an appropriate speed, it should be possible to track zebrafish larvae as well. Both organisms are therefore amenable to studies in virtual environments of the type demonstrated here. The system's high performance recentering capability could also be employed to target a laser beam for photostimulation or uncaging of small molecules in freely moving individuals of these species. When coupled with *z*-axis scanning, our system should facilitate imaging from neurons in freely moving fruit fly and zebrafish larvae.

## Methods

### Strains

*C. elegans*. Animals were cultivated under standard conditions [32], [33]. Transgenic strains were generated using the N2 wild-type strain. Calcium signals were recorded using the ratiometric probes as they are less sensitive to movement artifacts than non-ratiometric probes. The probes used were cameleon [7], TN-XL [8], and TN-XXL [9]. The following strains were used: XL158 *ntIS28*[*nmr-1::TN-XL*], XL161 *lin-15*; *cex-1::YC3.60*; *lin-15(+)*, XL177 *ntIS39*[*sra-11::YC3.60*] outcrossed 5×; XL178 *ntIs41*[*unc-17::sfTN-XXL*]; unc-122::dsRed. Optogenetic stimulation for creating virtual environments was performed using the strain XL195 (ntIs29[*nmr-1*::tdTomato]; ntIs27[*sra-6*::ChR2]; lite-1(ce314)). Animals were grown in presence or absence of 500 µM of all-trans-retinal, an essential co-factor for channelrhodopsin-2 function. *D. melanogaster*. Flies bearing transgenic ratiometric probe, UAS-YC3.60 [34] were crossed to the vesicular glutamate transporter enhancer trap line, OK371-Gal4 [35], to drive probe expression in all glutamatergic neurons. The resulting embryos were maintained in constant darkness at 30°C in the absence of food.

### Substrates

*C. elegans*. Worms were mounted for recording between a coverslip and a 3 mm thick agarose slab. The speed of locomotion and its waveform were similar to those of a worm crawling on standard agarose substrate (Figs. 4, 5, 6 and Movies S3, S4, S5). In Movie S1, a microfluidic chamber [12] was substituted for the agarose slab. *D. melanogaster*. Fly larvae were placed on a thin agarose layer on top of a coverslip.

### Recentering system

The main components of the recentering system were a commercially available motorized stage with its associated controller (MS-2000-PhotoTrack, Applied Scientific Instrumentation) and a four-quadrant photomultiplier tube (R8900U-00-M4, Hamamatsu) with its associated optics (PhotoTrack detector head, Applied Scientific Instrumentation). Recentering commands were computed by a dedicated circuit added to the controller by the manufacturer (Fig. S2). Briefly, analog signals from each of the four quadrant of the PMT were amplified and the values digitized by a microprocessor in the stage controller. Achieving high fidelity tracking required not only calculating the centering error, but also removing background signal and adjusting for balance errors caused by PMT channel gain variation and imperfect centering of the excitation light aperture and PMT aperture. Thus, the four values from the PMT quadrants were corrected by substracting background values before mulitplying by a fixed gain. The four background values were captured before each recording, whereas the four gain values were automatically set when tuning the tracking module during installation. The the background subtracted, and gain-corrected signals were then used to generate position error signals based on the skew in the light distribution across the PMT. Skew in the distribution of light falling on the PMT was calculated as
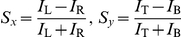
where *S_x_* and *S_y_* are skew in the *x* and *y* directions, *I*_L_ and *I*_R_ are the summed intensities on the left and right quadrants, and *I*_T_ and *I*_B_ are the summed intensities on the top and bottom quadrants. These skew values were multiplied by a second gain factor set by the experimenter based on the speed of the target. Scaled skew values were regulated the speed of the motorized stage. To reduce interference caused by background autofluorescence, *x* and *y* values below a user-defined threshold were ignored and a diaphragm could be adjusted to eliminate light from the edge of the field of view. When tracking red proxy targets, a dichroic mirror (565 dcsp, Chroma) was substituted for the 80/20 beam splitter. At the start of a recording session, the user brought the worm into the field of view of the 63× objective by manual control of the stage utilizing the live, wide-field image provided by the spotting camera, then pressed a key to activate the recentering mode of the stage controller.

### Calcium imaging

For optical recordings, worms (young adults) were mounted between a coverslip and a 3 mm thick agarose slab and placed on the stage of an inverted compound microscope (Zeiss Axiovert 135) fitted with a Zeiss plan-apochromat 63×, 1.4 NA oil immersion objective. Excitation light (436±10 nm) was provided by an X-cite 120 illuminator (Lumen Dynamics). A long-pass filter located above the 4× objective prevented excitation light from reaching the spotting camera. For ratiometry, images in cyan (480±15 nm) and yellow (535±20 nm) wavelength bands were projected side by side on the detector of the calcium imaging camera (ORCA AG, Hamamatsu) by means of a beam splitter (Dual-View, Optical Insights). Images were acquired using MetaVue software (version 6.2r2, Molecular Devices) and frames were taken at 20–40 Hz (corresponding to exposure times of 25–50 ms) with 4×4 spatial binning. Recording duration was limited to 90 s to avoid excessive photobleaching of the target.

Image stacks were processed off-line using custom analysis routines. Briefly, total intensity *I* in a region of interest (ROI) centered on the target neuron was measured in the 480 nm and 535 nm wavelength bands. A secondary ROI was drawn around the first one to measure the intensity of background fluorescence *I′*. The raw emission ratio was computed as *R* = (*I*_535_−*I′*_535_)/(*I*_480_−*I′*_480_)−0.65, where the latter term corrects for 480 nm channel bleed-through into the 535 nm channel. The emission ratio was corrected for photobleaching by fitting a single exponential function to the emission ratio trace and dividing the latter by the fitted function; thus all ratio changes were expressed in terms of changes in fluorescence, Δ*R*/*R*. To compensate for small changes in the position of the neuron in the field of view, the ROI was repositioned by centering it on the peak of intensity nearest to the coordinates of the pixel of highest intensity in the ROI in the previous frame. In the few instances when this automatic re-positioning of the ROI failed (because of fast movements of the neuron), the ROI was repositioned manually for at most 20 frames before automatic re-positioning resumed.

### Behavior

The instantaneous velocity vector was calculated by measuring the displacement of neighboring points in the *x-y* trajectory of the tracking target and dividing the sample interval. Velocity was smoothed by projecting the instantaneous velocity on its running vector average over a 1 s window. The animal's initial locomotory state (forward vs. reverse) was determined by visual inspection of the whole-animal video. States at subsequent time points were deduced automatically by custom software (Igor, Wavemetrics) that detected state transitions defined as changes in heading greater than 90° in the smoothed velocity vector. In side-by-side tests, we found that this method identified the beginning and end of each state more precisely than visual inspection of the video. The bending angle of the body wall *θ* was defined as the angle between the line segments formed by three points on the extracted outline of the animal: the center point of the tracking system and the two points located at a radial distance of 20 pixels (∼80 µm) from the center point as shown in Fig. 6a2. This angle was obtained from each image in the movies recorded by the spotting camera and automatically computed using custom software (Igor, Wavemetrics).

### Virtual environments via optogenetic stimulation

The position of the animal was detected by recording the stage position while a red target (*nmr-1*::tdtomato) was tracked. The target was excited by continuous 554 nm illumination focused through the 63× objective below the preparation (Fig. 1). The position of the animal was used to trigger a 445 nm light source focused through the low-power objective attached to the spotting camera.

## Supporting Information

Figure S1 **Step response performance test.** A fluorescent particle was placed 80 µm from the center of the field of view, and the tracking mode was engaged. Stage position is reported by the stage controller at the servo-loop rate, every 0.5 ms. The position of the stage is plotted over time for four different gain settings. Visual inspection of the curves indicates that the stage starts moving within approximately 2 ms, and centers the target in less than 30 ms, which is within the range of normal exposure times (25–50 ms). Data were obtained using a stage with a top speed of 13 mm/s.
(TIF)

Figure S2 **Block diagram of the tracking module.** Analog signals from the four PMT quadrants (a–d) were amplified and digitized. Light intensity values for the four quadrants were individually subjected to a background substraction and a gain compensation. The four corrected intensity values A–D were used to compute *S_x_* and S*_y_*, the skew in the distribution of light falling on the PMT in the *x* and *y* directions (see Methods). These skew values were multiplied by an user-defined gain to regulate stage speed and direction.
(TIF)

Movie S1 Video of a larval *C. elegans* crawling at high speeds in a fluid-filled matrix created in a microfluidic chamber. The animal was tracked by recentering its pharynx, which was labeled with the fluorescent protein tdTomato under the control of the *myo-2* promoter. The lower panel shows velocity versus time with a cursor indicating the velocity at the time of the current frame in the movie.
(M4V)

Movie S2 Video of a *Drosophila* larva crawling at speeds up to 500 µm/s on an agar surface. The animal was tracked by recentering glutamatergic neurons, which were labeled with the fluorescent indicator YC3.60. The lower panel shows velocity versus time with a cursor indicating the velocity at the time of the current frame in the movie.
(MOV)

Movie S3 Simultaneous recording of the activity of the command neuron AVA and the locomotory behavior of a freely moving animal. Upper left: video output of the spotting camera. The bright ring is reflectance from the 63× objective beneath the preparation. Upper right: *x*-*y* trajectory of the tracking target with a cursor indicating the *x*-*y* location at the time of the current frame in the movie. Lower left: video output of the calcium imaging camera. Lower right: time course of the AVA emission ratio (upper trace) and velocity (lower trace) with a cursor indicating the ratio and velocity at the time of the current frame in the movie (blue, forward; red, reverse).
(M4V)

Movie S4 Simultaneous recording of the activity of the command neuron AVB and the locomotory behavior of a freely moving animal. Upper left: video output of the spotting camera. The bright ring is reflectance from the 63× objective beneath the preparation. Upper right: *x*-*y* trajectory of the tracking target with a cursor indicating the *x*-*y* location at the time of the current frame in the movie. Lower left: video output of the calcium imaging camera. Lower right: time course of the AVB emission ratio (upper trace) and velocity (lower trace) with a cursor indicating the ratio and velocity at the time of the current frame in the movie (blue, forward; red, reverse).
(M4V)

Movie S5 Simultaneous recording of the activity of the motor neurons VA4 and VB5 and the locomotory behavior of a freely moving animal. Upper left: video output of the spotting camera. Red lines show the line segments that define the bending angle *θ.* Upper right: *x*-*y* trajectory of the tracking target with a cursor indicating the *x*-*y* location at the time of the current frame in the movie. Lower left: video output of the calcium imaging camera. Lower right: time course of the motor neuron emission ratios (upper traces) and bending angle (lower trace) with a cursor indicating the ratio and angle at the time of the current frame in the movie (blue, forward; red, reverse).
(M4V)

Table S1 Summary of motoneuron recordings showing the number of instances in which a motor neuron of the indicated type was recorded and the number of instances in which it exhibited ratio amplitude modulations that were correlated with dorsoventral undulations. Recordings from motor neurons that could not be positively identified are included for completeness. Motoneurons that did not exhibit activity correlated with dorsoventral undulations were flat.
(DOC)
