# Supplementary figures and images for: An Image-Free Opto-Mechanical System for Creating Virtual Environments and Imaging Neuronal Activity in Freely Moving Caenorhabditis elegans
Source: PLoS One. 2011 Sep 28;6(9):e24666. doi: 10.1371/journal.pone.0024666 (PMC3182168; doi:10.1371/journal.pone.0024666)

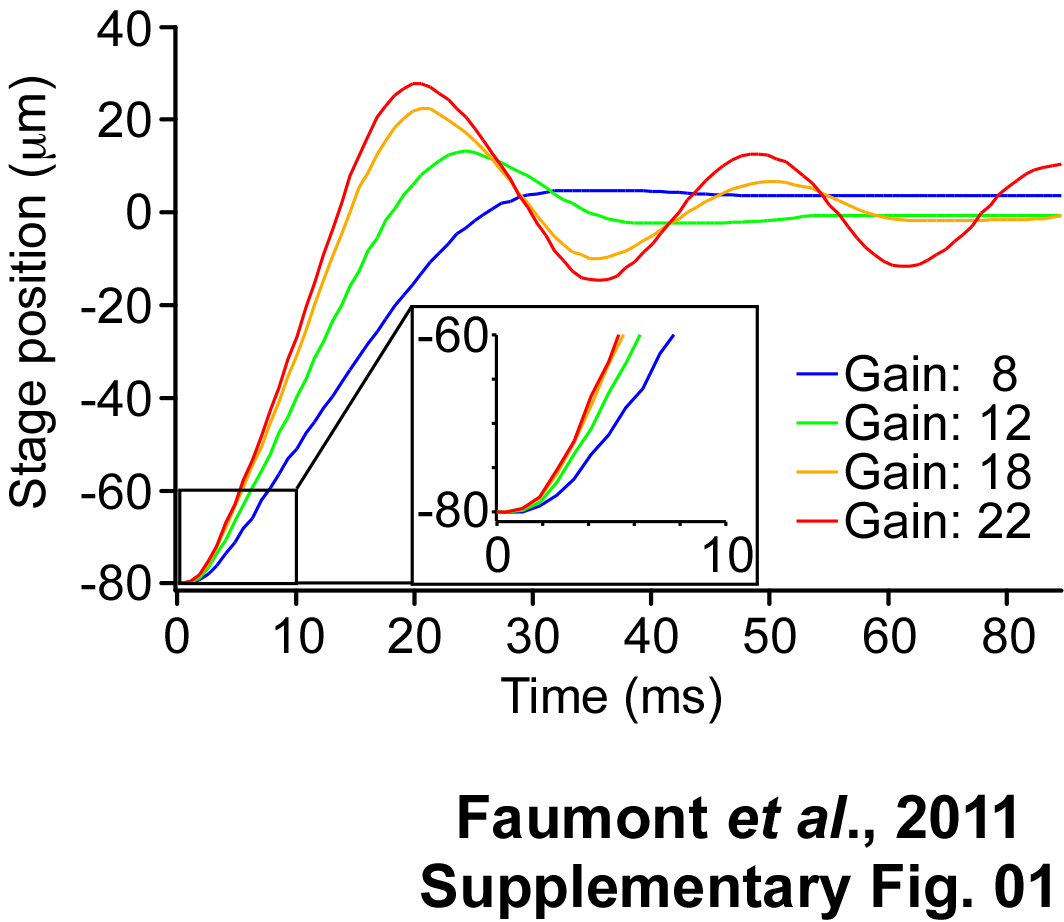

Supplement: Figure S1 — Step response performance test. A fluorescent particle was placed 80 µm from the center of the field of view, and the tracking mode was engaged. Stage position is reported by the stage controller at the servo-loop rate, every 0.5 ms. The position of the stage is plotted over time for four different gain settings. Visual inspection of the curves indicates that the stage starts moving within approximately 2 ms, and centers the target in less than 30 ms, which is within the range of normal exposure times (25–50 ms). Data were obtained using a stage with a top speed of 13 mm/s. (TIF) [file pone.0024666.s001.tif]

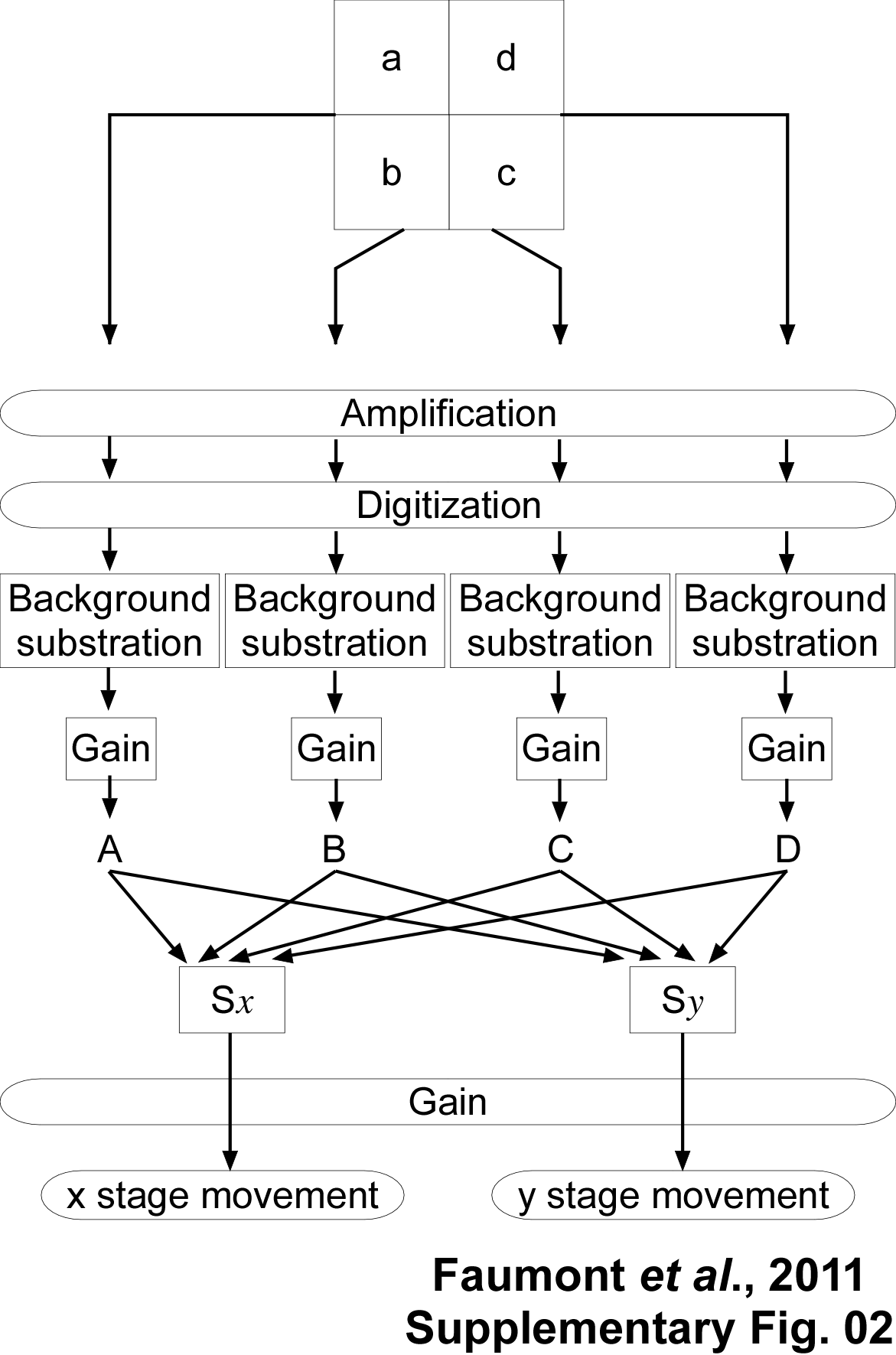

Supplement: Figure S2 — Block diagram of the tracking module. Analog signals from the four PMT quadrants (a–d) were amplified and digitized. Light intensity values for the four quadrants were individually subjected to a background substraction and a gain compensation. The four corrected intensity values A–D were used to compute Sx and Sy, the skew in the distribution of light falling on the PMT in the x and y directions (see Methods). These skew values were multiplied by an user-defined gain to regulate stage speed and direction. (TIF) [file pone.0024666.s002.tif]
