# Supplementary material for: An Image-Free Opto-Mechanical System for Creating Virtual Environments and Imaging Neuronal Activity in Freely Moving Caenorhabditis elegans
Source: PLoS One. 2011 Sep 28;6(9):e24666. doi: 10.1371/journal.pone.0024666 (PMC3182168; doi:10.1371/journal.pone.0024666)
Supplement: Table S1 — Summary of motoneuron recordings showing the number of instances in which a motor neuron of the indicated type was recorded and the number of instances in which it exhibited ratio amplitude modulations that were correlated with dorsoventral undulations. Recordings from motor neurons that could not be positively identified are included for completeness. Motoneurons that did not exhibit activity correlated with dorsoventral undulations were flat. (DOC) [file pone.0024666.s008.doc]

| Motoneuron type | Number of motor neurons recorded | Number of motor neurons with activity correlated with undulations |
| --- | --- | --- |
| VA | 9 | 6 |
| DA | 11 | 9 |
| VB | 7 | 6 |
| DB | 5 | 2 |
| VC | 5 | 3 |
| AS | 2 | 2 |
| Non-identified | 12 | 7 |

**Supporting table 1**. Summary of motoneuron recordings showing the number of instances in which a motor neuron of the indicated type was recorded and the number of instances in which it exhibited ratio amplitude modulations that were correlated with dorsoventral undulations. Recordings from motor neurons that could not be positively identified are included for completeness. Motoneurons that did not exhibit activity correlated with dorsoventral undulations did not display any activity.
